# Delivery in a Pregnant Woman With Pulmonary Arterial Hypertension (PAH): A Case Report

**DOI:** 10.7759/cureus.87565

**Published:** 2025-07-08

**Authors:** Szymon Paruszewski, Jacek Tatur, Bronislawa Pietrzak

**Affiliations:** 1 Department of Medicine, Medical University of Warsaw, Warsaw, POL; 2 Department of Obstetrics and Perinatology, National Medical Institute of the Ministry of Interior and Administration, Warsaw, POL

**Keywords:** atrial septal defect secundum, cesarean section (cs), high-risk pregnancy, severe pulmonary arterial hypertension, wearable cardioverter defibrillator

## Abstract

Cardiovascular diseases in pregnant women are a challenge in pregnancy and perinatal care, representing a significant cause of perinatal death among this population. In this case report, we describe a 41-year-old pregnant woman with atrial septal defect (ASD) type II, 30 mm in size, and secondary pulmonary arterial hypertension (PAH), who was admitted to the Department of Obstetrics and Perinatology for delivery at 35+5 weeks of her first pregnancy. During the first trimester, the patient's attending cardiologist decided to use a wearable cardioverter defibrillator on the patient until the end of the pregnancy and postpartum. At 36+0 weeks of pregnancy, the pregnancy was finished by elective cesarean section by an experienced obstetric and anesthetic team in a cardiosurgery operating room with extracorporeal membrane oxygenation (ECMO) access. The patient remained hemodynamically stable in time, and after the procedure, the newborn received 10 pt. Apgar. After the procedure and during the hospitalization until hospital discharge, the patient did not require emergency interventions. In conclusion, women with PAH should be advised against becoming pregnant, but if a woman becomes pregnant and does not consent to terminate the pregnancy, advanced multidisciplinary care should be provided.

## Introduction

Cardiovascular diseases in pregnant women are a challenge in pregnancy and perinatal care, representing a significant cause of perinatal deaths among this population in developed countries nowadays [1,2]. One of these conditions is pulmonary arterial hypertension (PAH), defined as abnormally high mean pulmonary arterial pressure (mPAP) >20 mmHg at rest [3], occurring in 9.7/100,000 pregnant women [4]. PAH carries a high risk of maternal mortality estimated at 30-56%, and this is dependent on the severity of the disease; severe PAH carries an approximately 20 times higher risk of maternal death than mild to moderate PAH, as well as a high risk of fetal mortality [2,4]. Pregnancy mortality is caused by an increase in plasma volume during pregnancy, right ventricular afterload, and end-diastolic volume, leading to right ventricular failure [5]. The risk is particularly high during labor and the postpartum period when the patient may be exposed to pain, acidosis, hypoxia, blood loss, hormonal changes, and the need for a rapid cardiovascular adaptation following the end of pregnancy resulting from utero-placental autotransfusion and reduced large vessel compression [6,7]. However, hemodynamic changes during pregnancy occur as early as 6-8 weeks, which can lead to the rapid deterioration of right ventricular function in early pregnancy [8]. Therefore, given the limited diagnostic and therapeutic options during pregnancy, all current guidelines strongly advise against pregnancy and recommend an effective method of contraception in women of reproductive age with PAH; if this fails, early termination of pregnancy is recommended [7,9].

PAH can be caused by heart, lung, or pulmonary vascular disease. Among the subtypes, PAH associated with congenital heart disease is distinctive, where the mortality rate of pregnant women, despite a decline over the years, remains high accounting for approximately 28% [2,10]. One of the congenital heart defects is an atrial septal defect (ASD), characterized by a slow clinical progression where most patients do not show clinical symptoms, which contributes to late diagnosis and the development of complications of the right part of the heart and pulmonary circulation overload due to left-to-right leakage, such as arrhythmias, right ventricular failure, pulmonary embolism, and, in a subset of patients, PAH. Pregnancy-induced cardiovascular changes can lead to a response of the "previously damaged" pulmonary arteries in the presence of congenital heart disease with left-to-right shunt, leading to right ventricular overload and, in the worst case, leading to the reversal of the shunt and the development of Eisenmenger's syndrome, which shows a high maternal mortality rate and frequent clinical deterioration after delivery [6].

## Case presentation

A 41-year-old pregnant woman with ASD type II and PAH secondary to congenital heart disease, in her first pregnancy, was admitted at 35+5 weeks of pregnancy in 2024 to the Department of Obstetrics and Perinatology. The patient was transferred from a monospecialty obstetrics hospital due to the multispecialty profile of our hospital to increase her safety.

The patient's ASD type II was discovered in 2018 during diagnostic tests after a sudden collapse. No worrying symptoms had previously been observed, and the patient was in a good general condition. She was scheduled for surgery to close the defect due to its significant size (approximately 30 mm), but the surgery date was postponed due to the COVID-19 pandemic; in June 2023, a follow-up echocardiogram (ECHO) revealed PAH in the patient. The woman became pregnant in January 2024; due to her condition, a termination of pregnancy was proposed, to which she did not agree. Due to the severity of the disease in the first trimester and the patient's lack of consent to terminate the pregnancy, the patient's attending cardiologist decided to use a wearable cardioverter defibrillator on the patient until the end of the pregnancy and the postpartum period. The wearable cardioverter defibrillator allowed continuous remote monitoring of the patient's electrocardiogram (ECG) by the cardiologist.

On the day of admission to the department, the patient presented with normal blood pressure, saturation, and heart rate values. She denied the symptoms such as dyspnoea and chest pain; on physical examination, discrete cyanosis of the lips was noted. Results of laboratory tests on the day of admission are mentioned in Table 1. The patient was taking sustained-release metoprolol 75 mg in the morning and 25 mg in the evening, amiloride 2.5 mg with hydrochlorothiazide 25 mg every other day, and potassium and magnesium with vitamin B6. Antithrombotic prophylaxis with low-molecular-weight heparin was included due to the high thromboembolic risk.

**Table 1:** Results of laboratory tests on the day of admission to the clinic. NT-proBNP: N-terminal pro-B-type natriuretic peptide; eGFR: estimated glomerular filtration rate; sFlt-1/PlGF: soluble fms-like tyrosine kinase 1/placental growth factor; LDH: lactate dehydrogenase; ALT: alanine aminotransferase The norm depends on the week of pregnancy; for our patient (after the 34th week of pregnancy), sFlt-1/PlGF is <85 which means low risk of pre-eclampsia.

Laboratory test	Patient values	Reference range
NT-proBNP	515 pg/ml	<125 pg/ml
High-sensitivity troponin I	53.1 pg/ml	<15.6 pg/ml
Creatinine	0.81 mg/dl	0.5-0.9 mg/dl
eGFR	93 ml/min/1.73 m^2^	>90 ml/min/1.73 m^2^
Hemoglobin	10.8 g/dl	11-14 g/dl
White blood cells	9.37×10³/µl	4.5-10×10³/µl
Platelets	193×10³/µl	150-400×10³/µl
sFlt-1/PlGF ratio	51.08 pg/ml	-
Uric acid	10.8 mg/dl	2.4-5.7 mg/dl
LDH	226 U/l	135-214 U/l
Fibrinogen	625 mg/dl	170-420 mg/dl
ALT	17 U/l	5-31 U/l

The pregnancy was complicated by gestational diabetes mellitus treated with diet (GDM1), detected by an oral glucose tolerance test in the second trimester of pregnancy, and nicotinism in early pregnancy. The patient menstruated irregularly before pregnancy. The date of the patient's last menstrual period was January 15, 2024, and the date of birth according to the Naegele rule was October 21, 2024, and, according to the first-trimester ultrasound (USG), October 16, 2024. The patient had negative serum tests for toxoplasmosis, other agents, rubella, cytomegalovirus, and herpes simplex (TORCH) microorganisms, a normal level of glycemia in the first trimester, and a normal cytology of the cervix result. Vaginal and rectal bacterial cultures for *Streptococcus agalactiae* type B were positive. On the day of admission, a USG showed a fetus in the cephalic position with normal biometry and vascular flow (estimated fetal weight (EFW)-Hadlock II: 2372 g) and normal amniotic fluid volume (amniotic fluid index (AFI): 22.43 cm; norm 5-24 cm). There were no abnormalities in obstetric examination or cardiotocogram (CTG). In laboratory tests, the soluble fms-like tyrosine kinase 1/placental growth factor (sFlt-1/PlGF) ratio on the day of admission was 51.08 pg/ml and in a repeat test on the following day 82.54 pg/ml.

At 36+0 weeks of pregnancy, after prior consultation with a cardiac anesthetist, in the conditions of a cardiosurgery operating room with available medical equipment for extracorporeal membrane oxygenation (ECMO), the pregnancy was finished by elective cesarean section by an experienced obstetric team, with an anesthetic team experienced in participating in cardiac surgery. The patient was given subarachnoid anesthesia, and the cesarean section procedure using the Pfannenstiel and Misgav-Ladach methods proceeded without complications. The newborn boy delivered from the uterus weighed 2500 g, was 50 cm long, and received 10 pt. Apgar. One gram of tranexamic acid was administered. During the procedure, the patient's heart rate was 115-125/min, her blood pressure was 140/80-170/80 mmHg, the heart rhythm was sinus and regular, and no vasoconstrictive drugs were used. At the end of the procedure, the patient remained hemodynamically stable, with a contracted uterus, and was transferred for further treatment in the postoperative unit of the Department of Anesthesiology and Intensive Care for 24 hours, after which she was transferred to the Department of Obstetrics and Perinatology. After surgery and during the stay until hospital discharge, the patient did not require emergency interventions.

**Figure 1:**
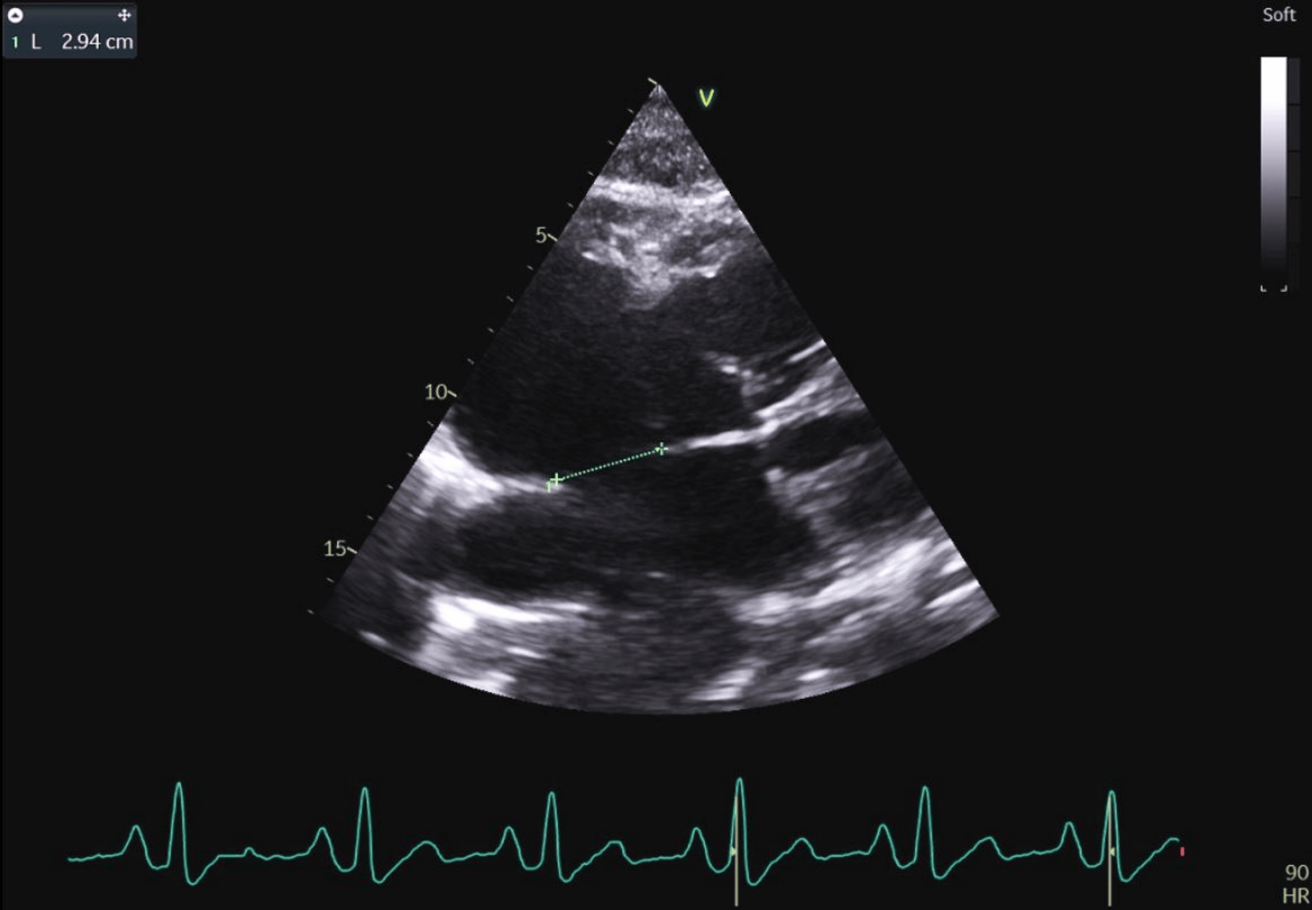
Cross-section through the atria demonstrating an atrial septal defect type II 29.4 mm in size.

**Figure 2:**
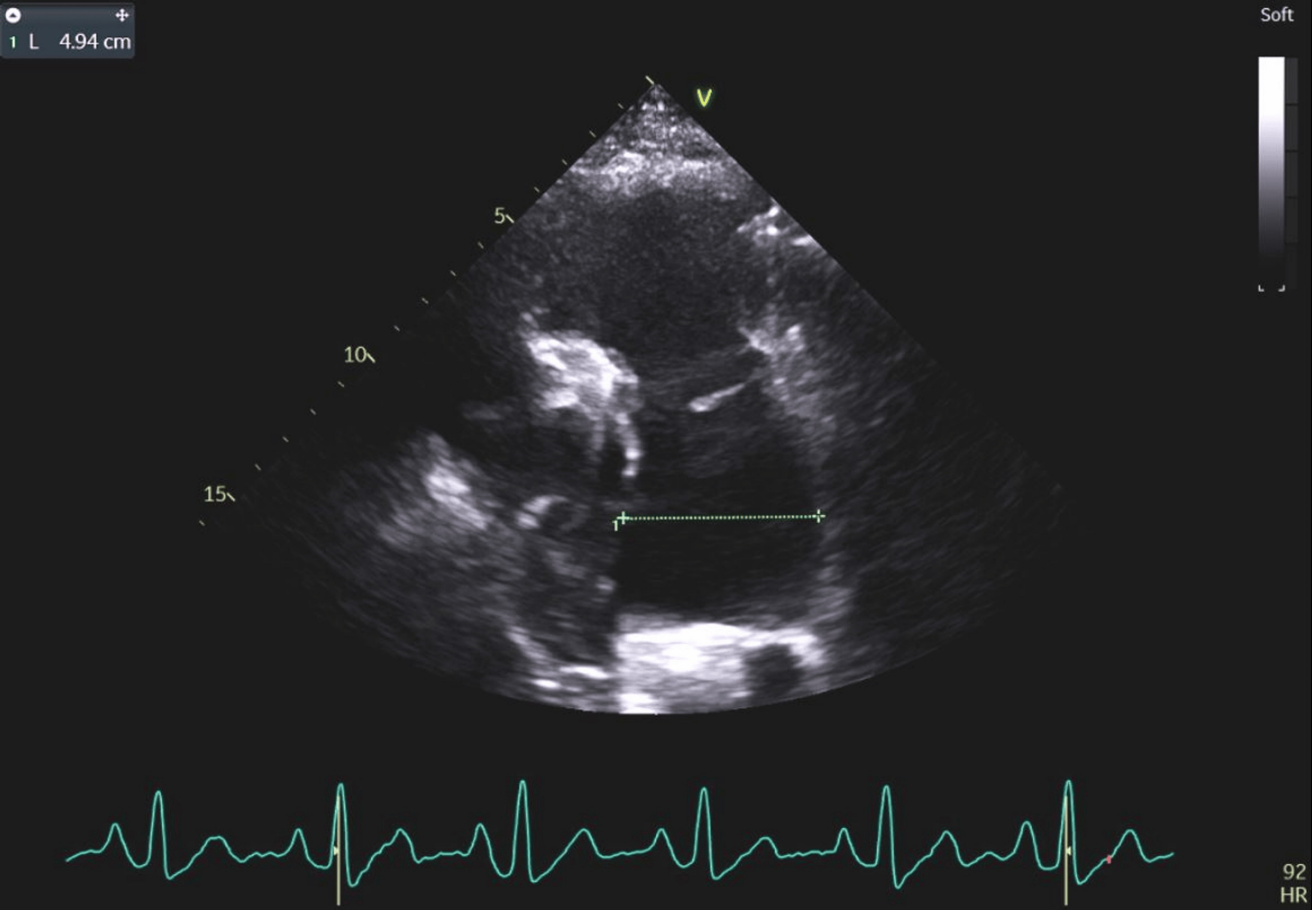
Aneurysmal dilatation of the pulmonary trunk (49.4 mm with norm <25 mm) secondary to right ventricular volume overload caused by left-to-right leakage through the atrial septum.

**Figure 3:**
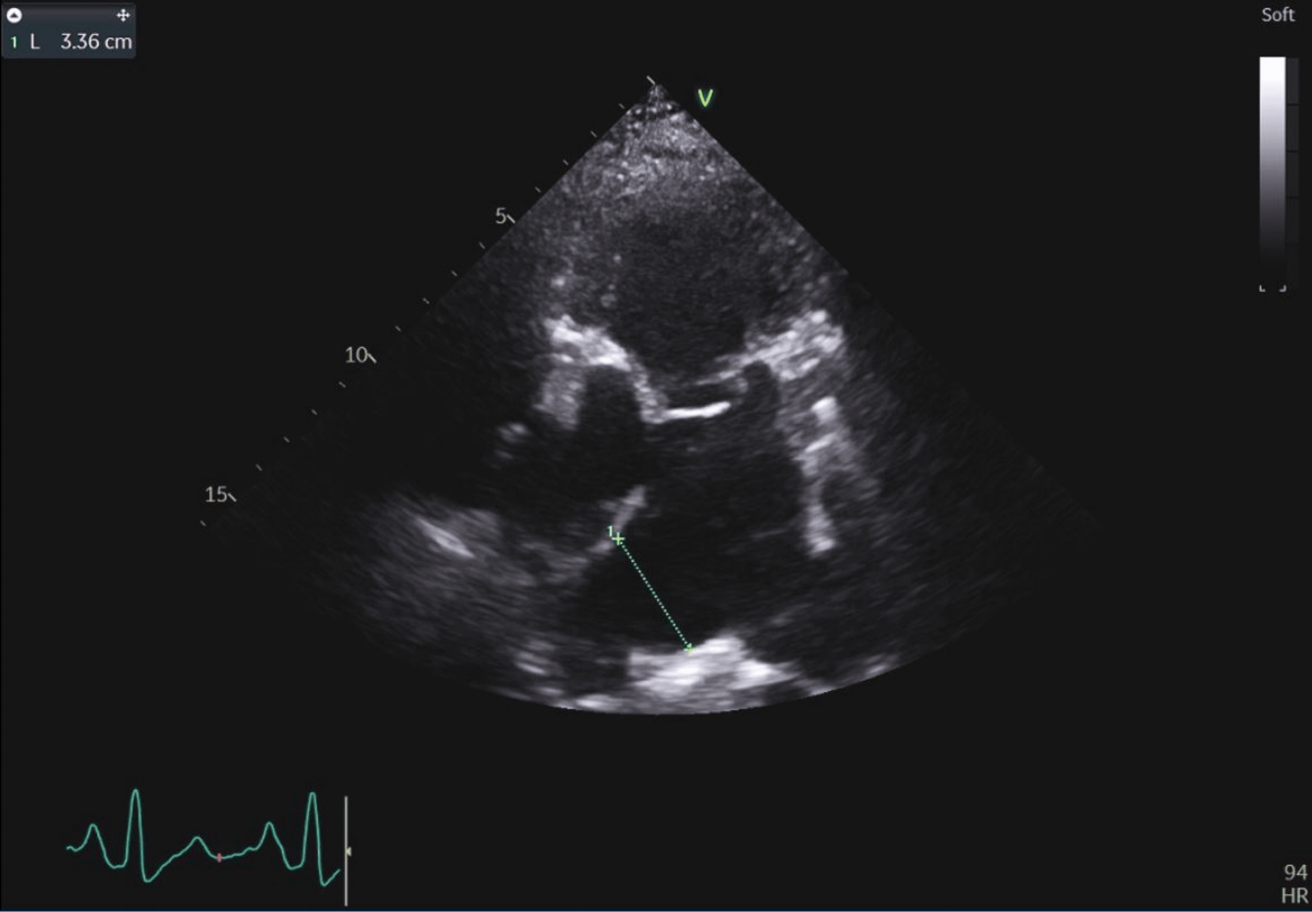
Right pulmonary artery dilatation (33.6 mm with norm <20 mm) secondary to right ventricular volume overload caused by left-to-right leakage through the atrial septum.

**Figure 4:**
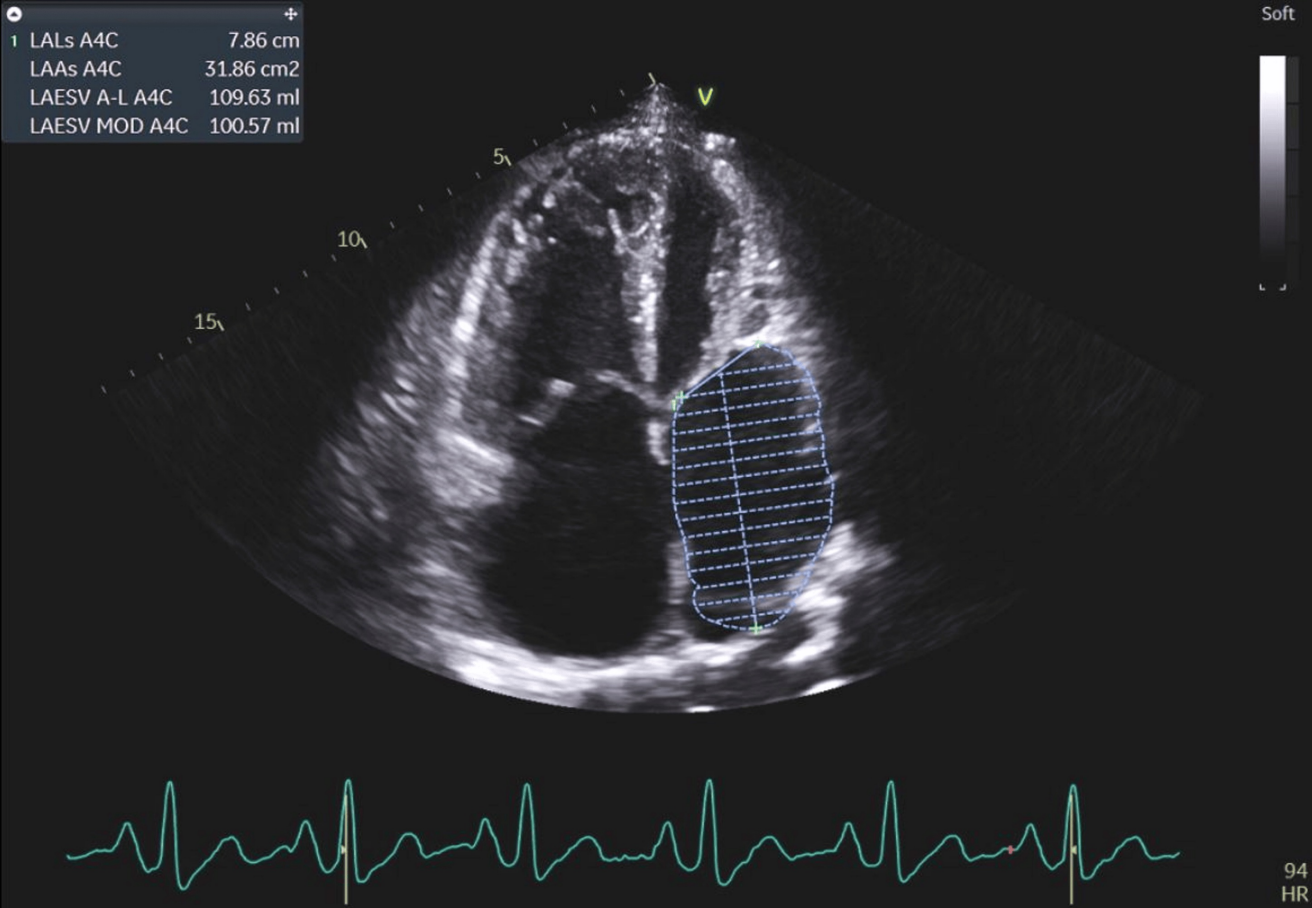
The patient's left atrial enlargement secondary to coexisting minor stenosis and mitral valve regurgitation, both of which cause chronic left atrial volume-pressure overload. LALs A4C: left atrial length, systolic, apical 4 chamber; LAAs A4C: left atrium area, systolic, apical 4 chamber; LAESV A-L A4C: left atrial end-systolic volume, area-length, apical 4 chamber; LAESV MOD A4C: left atrial end-systolic volume, method of discs, apical 4 chamber

**Figure 5:**
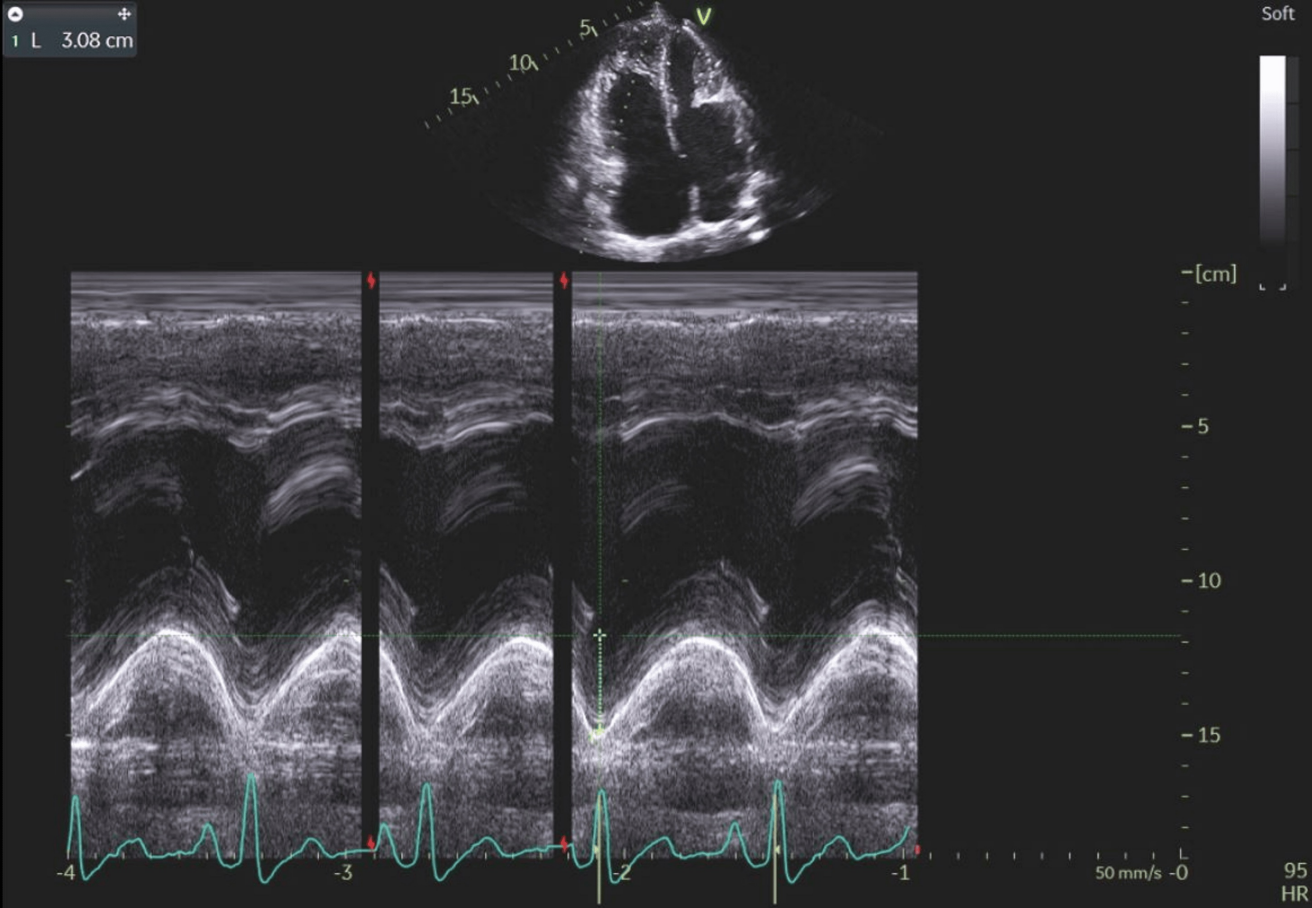
M-mode imaging of the apical four-chamber cross-section showing a TAPSE value of 3.08 cm, which indicates very good right ventricular systolic function and may indirectly suggest volume overload of the right ventricle. TAPSE: tricuspid annular plane systolic excursion

**Figure 6:**
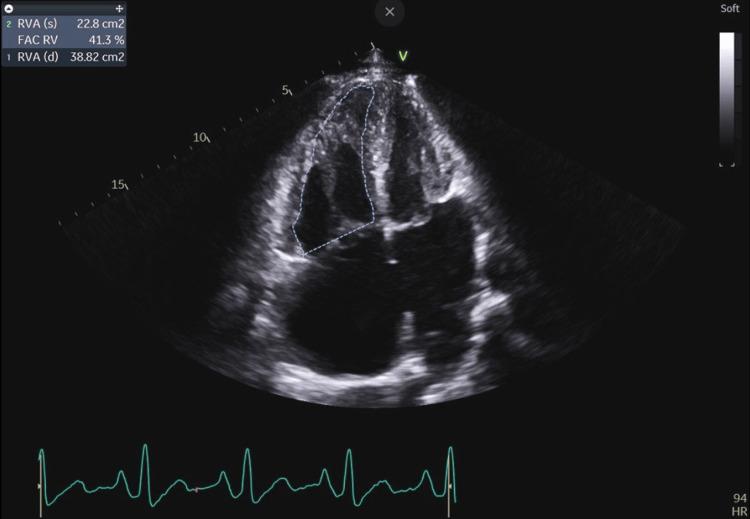
Enlargement of the right heart cavities with preserved right ventricular systolic function (FAC RV 41.3%) was visualized, suggesting volume overload. RVA (s): right ventricular area, systole; RVA (d): right ventricular area, diastole; FAC RV: fractional area change of right ventricle

**Figure 7:**
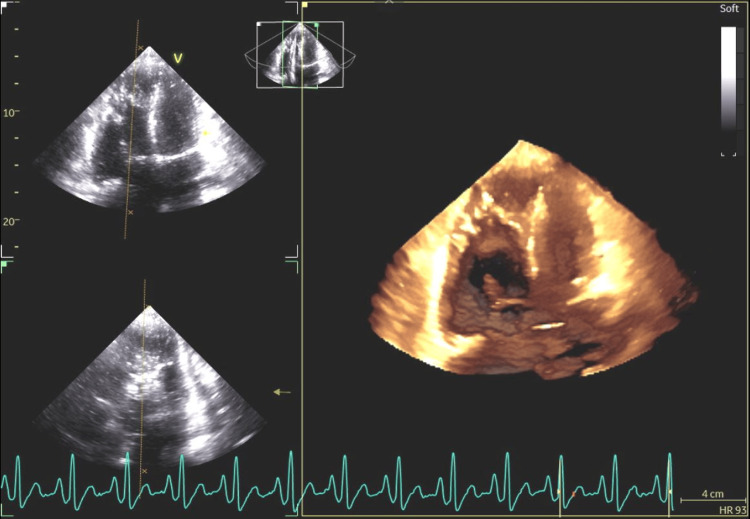
The three-dimensional reconstruction showing a four-chamber cross-section through the heart, showing the atrial septum, in which an atrial septal defect type II is present.

**Figure 8:**
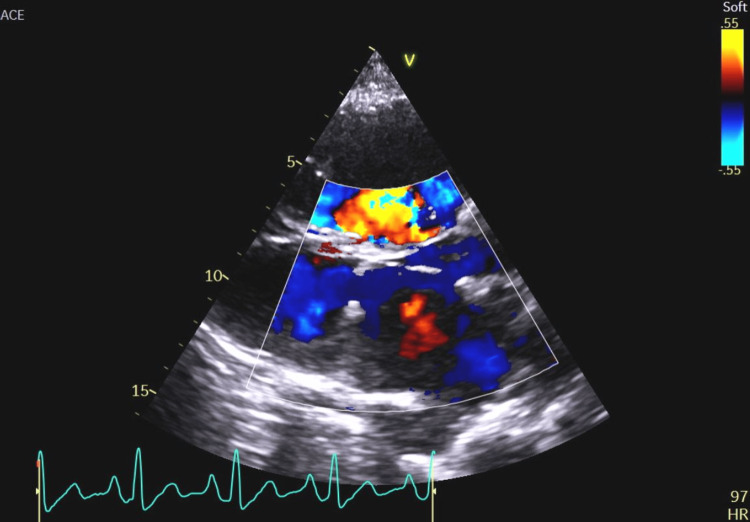
Cross-section through the atria demonstrating left-to-right leakage through the atrial septum.

**Figure 9:**
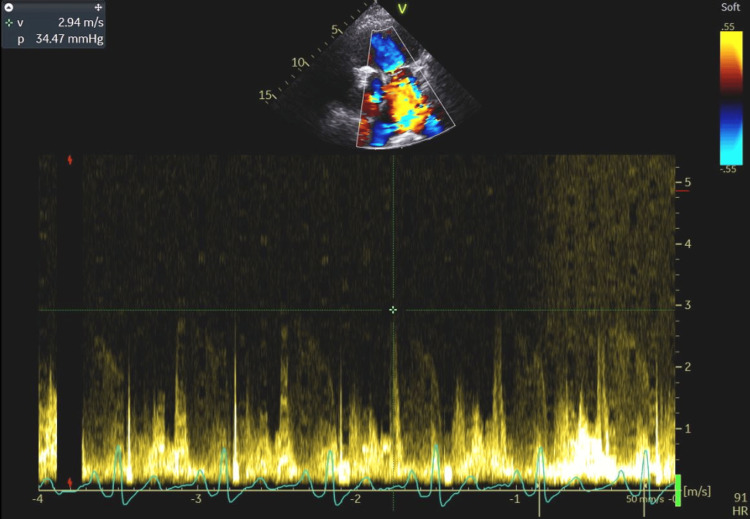
A flow velocity of 2.94 m/s indicating that blood flows through the tricuspid valve faster than normal. Based on this, a pressure gradient of 34.47 mmHg was calculated, suggesting that pulmonary artery pressure is elevated.

**Figure 10:**
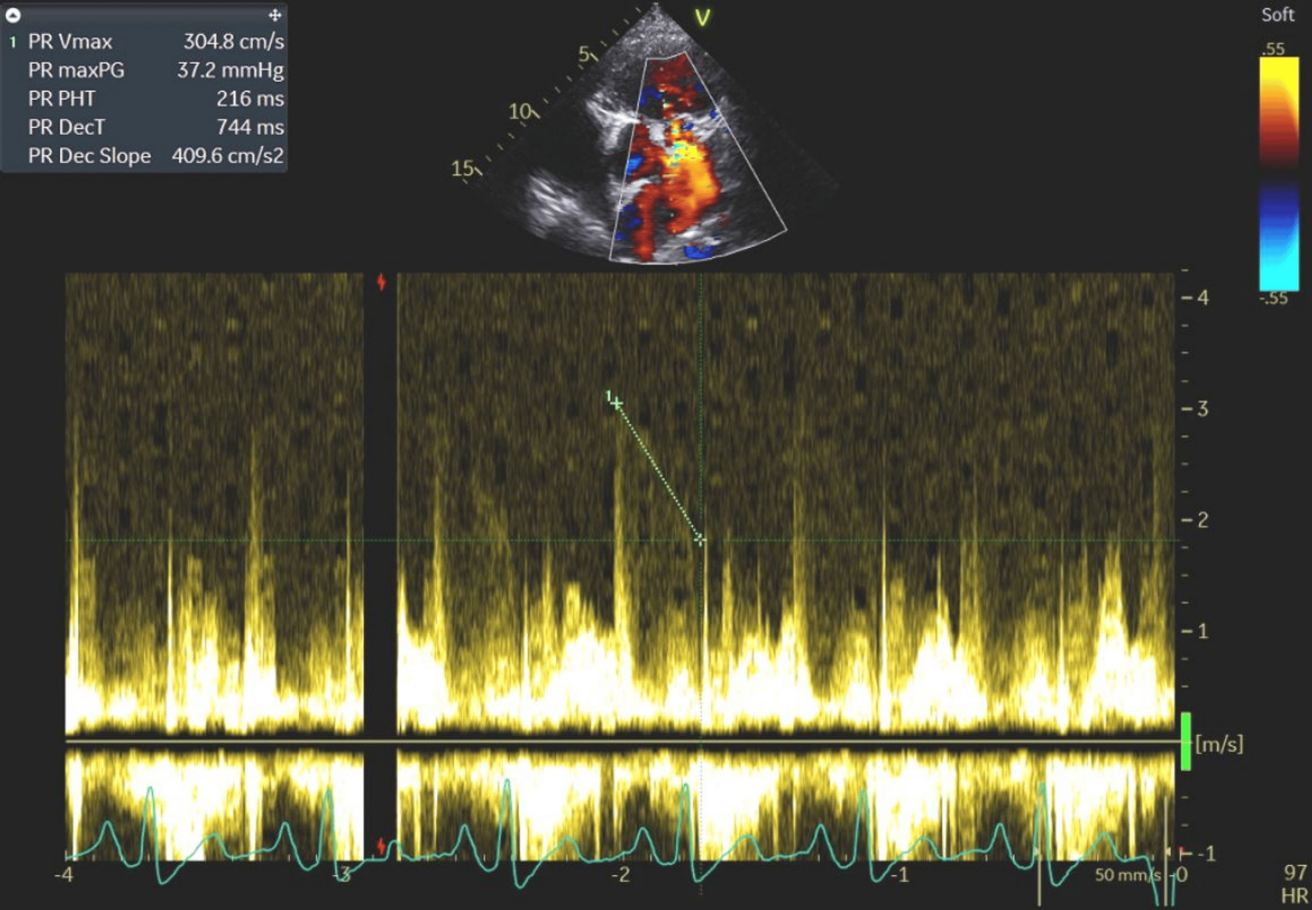
The high pulmonary regurgitation velocity (PR Vmax 3.05 m/s) and maximum pressure gradient of 37.2 mmHg, combined with a shortened half-time (PR PHT 216 ms) and steep deceleration wave slope (PR Dec Slope 409.6 cm/s²), indicating significant pulmonary hypertension, most likely secondary to chronic volume overload of the right heart. PR Vmax: pulmonary regurgitation maximum velocity; PR maxPG: pulmonary regurgitation maximum pressure gradient; PR PHT: pulmonary regurgitation pressure half-time; PR DecT: pulmonary regurgitation deceleration time; PR Dec Slope: pulmonary regurgitation slope of the deceleration

**Figure 11:**
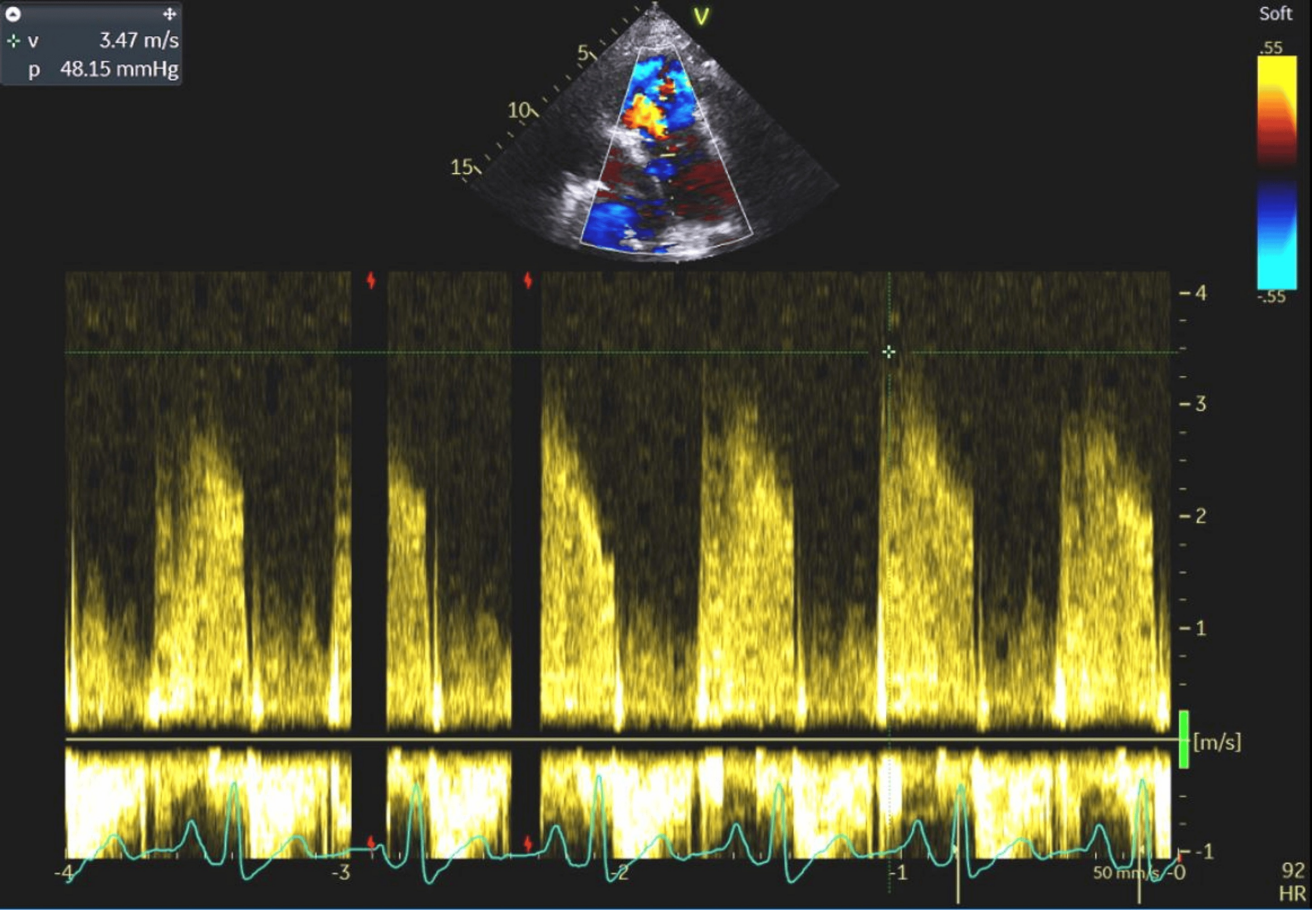
The measured tricuspid regurgitation velocity of 3.47 ms and pressure gradient of 48.15 mmHg indicating significant pulmonary hypertension, which may suggest chronic volume overload of the right heart.

**Figure 12:**
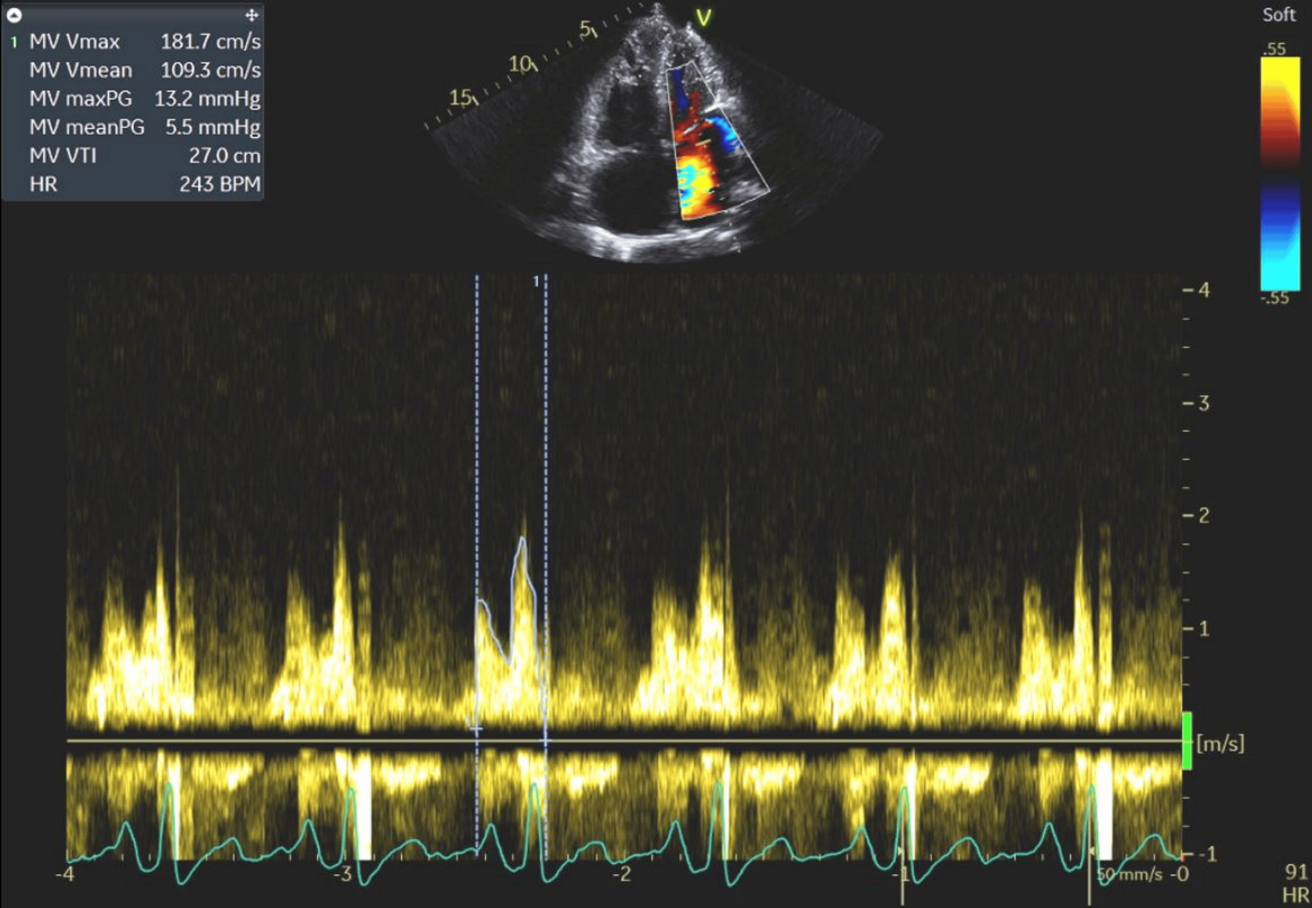
Significantly accelerated flow across the mitral valve (MV Vmax=181.7 cm/s; MV mean=109.3 cm/s) and elevated pressure gradients (maxPG=13.2 mmHg; meanPG=5.5 mmHg) were seen, indicating left ventricular volume overload. MV Vmax: mitral valve maximum velocity; MV Vmean: mitral valve mean velocity; MV maxPG: mitral valve maximum pressure gradient; MV meanPG: mitral valve mean pressure gradient; MV VTI: mitral valve velocity time integral; HR: heart rate

**Figure 13:**
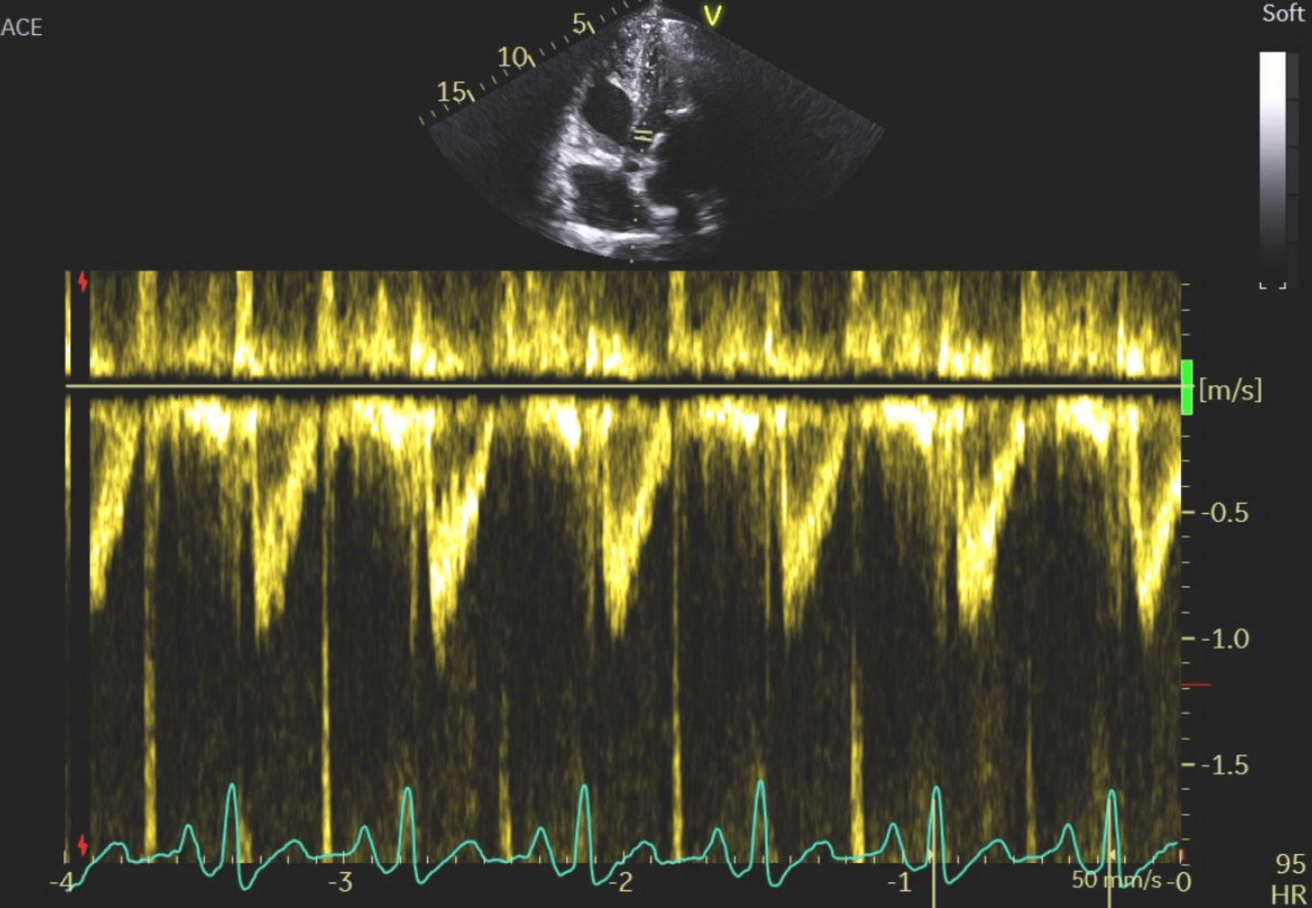
Normal systolic velocity S' of 16 cm/s indicating preserved right ventricular systolic function.

**Figure 14:**
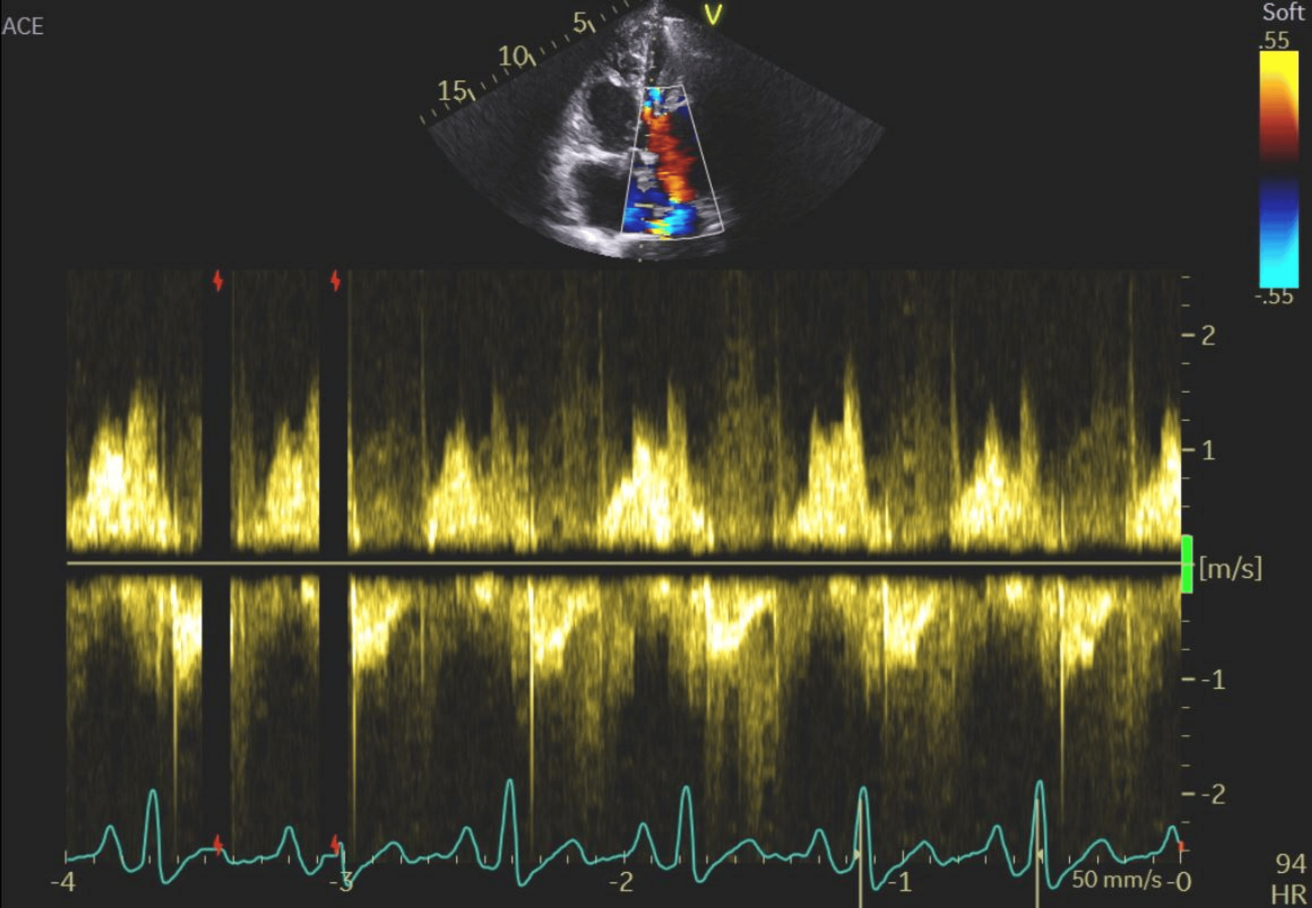
Spectral Doppler of flow across the mitral valve showing a biphasic flow with a dominant E-wave with a peak velocity of about 1.2 m/s and an A-wave of about 0.7 m/s, suggesting a normal or mildly increased diastolic gradient without features of significant left ventricular diastolic dysfunction.

**Figure 15:**
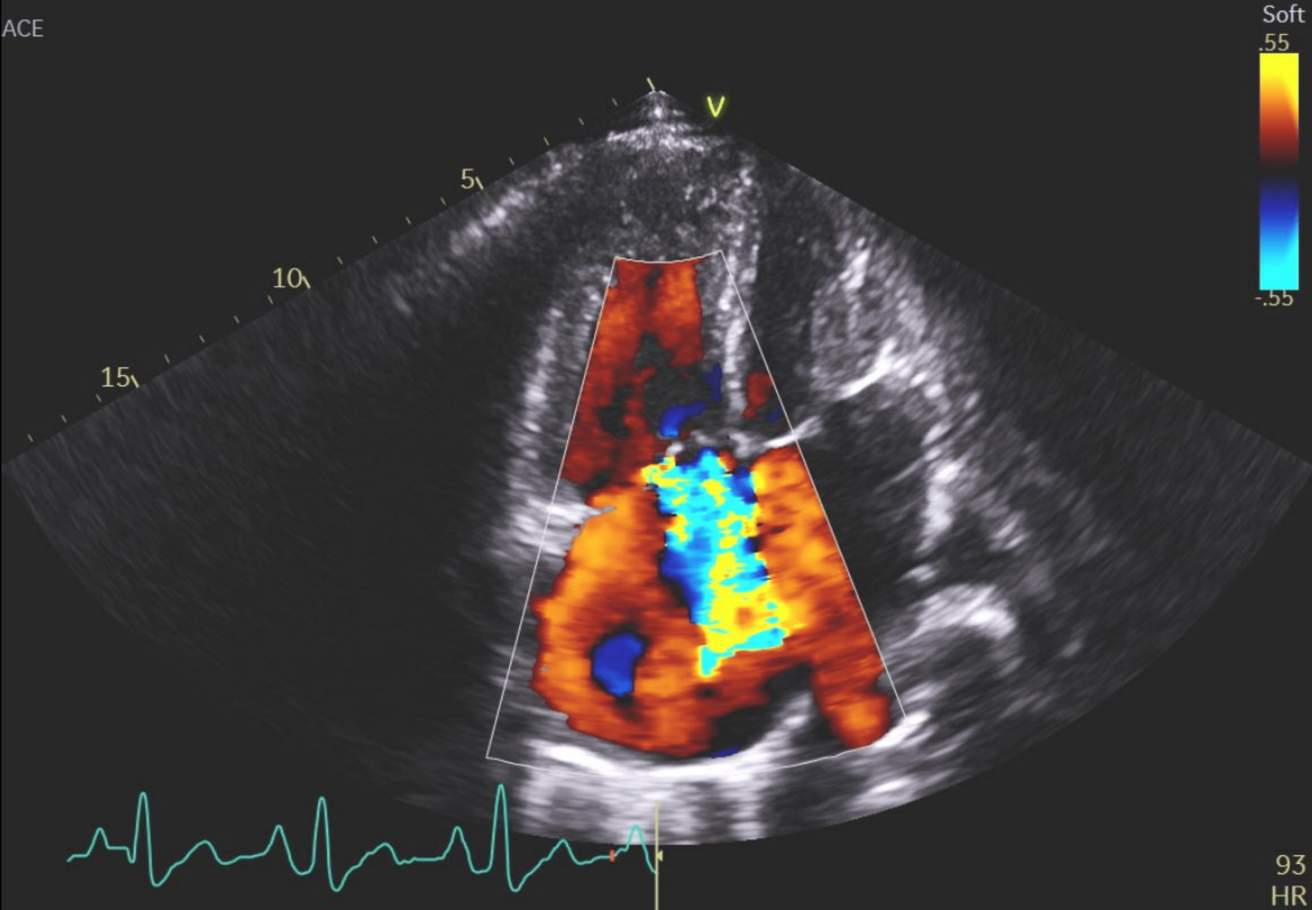
An echocardiographic image in the apical four-chamber view with color Doppler showing a left-to-right leak through the atrial septum.

In early puerperium, N-terminal pro-B-type natriuretic peptide (NT-proBNP) and high-sensitivity troponin I levels increased. The dynamics of the results of these cardiac laboratory tests are shown in Table 2. The patient showed no clinical signs of cardiorespiratory failure. On the fifth postpartum day, the patient underwent a follow-up ECHO revealing a significant dilatation of the right atrial and right ventricular cavities, dilated left atrial cavity, and small left ventricular cavity; normal left ventricular wall thickness and right ventricular hypertrophy of 9 mm were observed. The overall contractility of the left and right ventricles was assessed as good, with no obvious abnormalities. The mitral valve leaflets had slight degeneration, and slight stenosis and slight mitral regurgitation were observed. The aortic valve had no significant changes. Severe tricuspid regurgitation and features of PAH with flattening of the interventricular septum (right ventricular systolic pressure after accounting for pulmonary stenosis: 56 mmHg) were observed. The pulmonary valve leaflets had fibrosis, and slight stenosis and slight pulmonary regurgitation were observed. An aneurysmal dilated pulmonary trunk and dilated pulmonary arteries, 33-mm-wide on the right, were observed. The inferior vena cava had normal width and reduced respiratory variability. A common inferior vena cava and hepatic vein run-off was observed. The ASD type II did not change its diameter; it was approximately 30 mm, and a significant left-to-right leak was observed. The results of ECHO are summarized in Table 3 and are visible in Figures 1-15.

**Table 2:** The dynamics of results of cardiac laboratory tests. NT-proBNP: N-terminal pro-B-type natriuretic peptide

Laboratory test	Day of admission	Third postpartum day	Fourth postpartum day
NT-proBNP (<125 pg/ml)	515 pg/ml	1660 pg/ml	2609 pg/ml
High-sensitivity troponin I (<15.6 pg/ml)	53.1 pg/ml	63 pg/ml	50.4 pg/ml

**Table 3:** Echocardiographic results of the heart in early puerperium. LVEF: left ventricular ejection fraction; TAPSE: tricuspid annular plane systolic excursion; RV FAC: right ventricular fractional area change; Est RVSP: estimated right ventricular systolic pressure; RA press: right atrium pressure; MV: mitral valve; MV maxPG: maximum pressure gradient; MV meanPG: mean pressure gradient; TV: tricuspid valve; TV Vmax: peak velocity; VC: vena contracta; PISA: proximal isovelocity surface area; PV: pulmonary valve; PV Vmax: peak velocity; PV maxPG: maximum pressure gradient

Ventricular systolic function	Right atrium and ventricle	MV	TV	PV
LVEF: 60%	Est RVSP: 80.56 mmHg	MV maxPG: 13 mmHg	TV Vmax: 4.2 m/s	PV Vmax: 2.4 m/s
TAPSE: 30 mm	RVSP after accounting for pulmonary stenosis: 56 mmHg	MV meanPG: 5 mmHg	VC: 5 mm	PV maxPG: 24 mmHg
RV FAC: 41%	RA press: 10 mmHg	Regurgitation: low	PISA: 9 mm	Regurgitation: low
			Regurgitation: severe	

The patient was discharged home from the hospital on the sixth postpartum day with the standard recommendations from an obstetrician and, in addition, recommendations to maintain the prepartum drug treatment and to continue treatment under the care of a cardiologist. She, under the care of a cardiologist, wore a wearable cardioverter defibrillator for three months after delivery and did not experience any complaints related to the disease during this time. At 10 months postpartum, the patient is in good general condition, has no symptoms related to the disease, and awaits for the percutaneous closure of the ASD, and her son develops normally.

## Discussion

Women patients with PAH secondary to a congenital heart defect (particularly with Eisenmenger's syndrome) should be advised against becoming pregnant. During pregnancy, plasma volume increases, exacerbating left-to-right leakage through the ASD, but vascular resistance decreases, increasing the risk of leakage reversal and the development of Eisenmenger's syndrome, which results in a decrease in the arterial blood oxygen partial pressure; maternal mortality in Eisenmenger's syndrome is estimated at 30-60% and is associated with syncope, thrombosis, hypovolemia, hemoptysis, or pre-eclampsia [10,11]. Fetal or neonatal death ranges from 10% to 45%. The most commonly reported adverse events are fetal growth restriction and preterm birth [12,13].

## Conclusions

If a woman becomes pregnant and does not agree to terminate the pregnancy, appropriate cardiac, perinatal, postnatal, neonatal, and anesthetic care should be provided. The delivery should take place in the highest referral hospital centre where ECMO procedures and cardiac surgery, including heart transplantation, are available. Both vaginal delivery and cesarean section are possible; in the case of a vaginal delivery, the expulsion phase should be shortened as much as possible by a procedural vaginal delivery (using obstetric vacuum or obstetric forceps). Hypotension should be treated immediately with fluid therapy and vasoconstrictive drugs.

The above-described clinical case presents a situation in which the pregnant woman with PAH who did not consent to a termination of pregnancy, due to the medical team's preparation for the interdisciplinary management of the patient in the perinatal period, was able to give birth to a healthy child, and all measures were made to ensure her safety.
